# Numerical simulation and verification of rotor downwash flow field of plant protection UAV at different rotor speeds

**DOI:** 10.3389/fpls.2022.1087636

**Published:** 2023-01-26

**Authors:** Kun Chang, Shengde Chen, Meimei Wang, Xinyu Xue, Yubin Lan

**Affiliations:** ^1^ College of Electronic Engineering, College of Artificial Intelligence, South China Agricultural University, Guangzhou, China; ^2^ National Center for International Collaboration Research on Precision Agricultural Aviation Pesticides Spraying Technology, South China Agricultural University, Guangzhou, China; ^3^ Guangdong Laboratory for Lingnan Modern Agriculture, South China Agricultural University, Guangzhou, China; ^4^ Department of Mechanical Engineering, Anyang Institute of Technology, Nanjing Institute of Agricultural Mechanization, Ministry of Agriculture and Rural Affairs, Nanjing, China; ^5^ Nanjing Institute of Agricultural Mechanization, Ministry of Agriculture and Rural Affairs, Nanjing, China; ^6^ Department of Biological and Agricultural Engineering, Texas A&M University, College Station, TX, United States

**Keywords:** plant protection UAV, dynamic load effect, lattice Boltzmann method (LBM), particle image of velocity (PIV), the numerical simulation

## Abstract

In aerial spraying of plant protection UAVs, the continuous reduction of pesticides is an objective process. Under the condition of constant flight state (speed and altitude), the change of pesticide loading will inevitably lead to the shift of lift force and rotor speed generated by UAV rotor rotation, which will change the distribution of the rotor flow field and affect the effect of aerial spraying operation of plant protection UAV. Therefore, the rotor speed of UAV is taken as the research object in this paper, and the adaptive refinement physical model based on the Lattice Boltzmann Method (LBM) is used to numerically simulate the rotor flow field of the quadrotor plant-protection UAV at different speeds. A high-speed particle image velocimeter (PIV) was used to obtain and verify the motion state of the droplets emitted from the fan nozzle in the rotor flow field at different speeds. The results show that, with the increase of rotor speed, the maximum velocity and vorticity of the wind field under the rotor increase gradually, the top wind speed can reach 13m/s, and the maximum vorticity can reach 589.64*s* ^-1^. Moreover, the maximum velocity flow value is mainly concentrated within 1m below the rotor, and the maximum vorticity value is primarily concentrated within 0.5m. However, with the increase of time, the ultimate value of velocity and vorticity decreases due to the appearance of turbulence, and the distribution of velocity and vorticity are symmetrically distributed along the centre line of the fuselage, within the range of (-1m, 1m) in the X direction. It is consistent with the motion state of droplets under the action of the rotor downwash flow field obtained by PIV. The study results are expected to reveal and understand the change law of the rotor flow field of plant protection UAVs with the dynamic change of pesticide loading to provide a theoretical basis for the development of precise spraying operation mode of plant protection UAVs and improve the operation effect.

## Introduction

The application technology of plant protection unmanned aerial vehicle (UAV) has the advantages of high operation efficiency, low operation cost, and no limitation of operation geographical area and crop growth, which is one of the key technologies to realize the modernization of field management (Dongyan et al., 2014; Yong et al., 2017). Early domestic researchers initially focused on application operation parameters and droplets deposition (Yuan-yuan, 2013; Shuai, 2014). Since 2016, the research scope of plant protection UAV applications began to multiply and extend to other application objects. For example, the distribution of spraying effect in the citrus orchard was studied under different operating parameters (Pan and Qiang, 2016); Study on spraying corn with droplets using JF01-10 plant protection UAV in different growing stages (Zheng et al., 2017); Study on wheat scab control by using DJI T30 plant protection UAV (Tang et al., 2018). That can be seen that plant protection UAVs have been widely used in modern precision agriculture (Huang et al., 2013; Shahbazi et al., 2014; Xiongkui et al., 2017; Chen et al., 2022). In particular, the quadrotor plant protection UAV, the most important type of plant protection UAV, has been effectively applied to prevent and control diseases, insects and weeds in various countries (Jiyu et al., 2018; Wang et al., 2019; Zhan et al., 2022).

Generally, in terms of studying the effect of droplets deposition and distribution, field experiments mainly use materials such as water-sensitive papers, Mylar sheets, Petri dishes and polyethene wires to study the related parameters of wind field distribution characteristics (Shengde et al., 2016; Xiaonan et al., 2017; Wang et al., 2018; Wu et al., 2019). Through these materials, the droplets in the vertical and horizontal planes can be collected in space movement. Still, only the deposition effect of the pesticide droplets can be observed, and the deposition motion state and mechanism of the droplets can not be directly revealed. The rotor wind field generated by the plant protection UAV is the most critical factor affecting aerial spray droplets deposition and distribution characteristics in the gradual settlement of pesticide droplets under the rotor wind field (Songchao et al., 2015; Songchao et al., 2017). In recent years, some researchers have also used wireless wind sensor networks, ultrasonic anemometer arrays and tensiometers to measure the change of instantaneous wind field under the rotor to reveal the influence mechanism of the rotor downwash wind field on droplets deposition (Jiyu et al., 2014a; Zhang et al., 2016; Songchao et al., 2017; Wang et al., 2018; Tang et al., 2019; Wu et al., 2019). In the whole process of aerial spraying of plant protection UAV, with the continuous reduction of the pesticide in the pesticide box, its mission load parameters are always in a constant dynamic change process. Therefore, the effect of the rotor wind field of plant protection UAV under dynamic load is bound to differ (Jiyu et al., 2014a; Jiyu et al., 2014b; Shengde et al., 2016; Chen et al., 2017). Nevertheless, the above research is only focused on the hover state or a particular condition to test and analyze, and the change of pesticide load in the actual operation condition determines that the rotor wind field distribution is a process of continuous change. Therefore, there are many limitations; the above research results cannot directly reflect plant protection UAV’s rotor wind field distribution transformation in the whole operation process.

With the improvement of the computing power of computers and the gradual improvement of the theory of fluid mechanics, the cross combination of the two makes computational fluid dynamics(CFD) widely in-depth into various fields. Especially in the field of agriculture, computational fluid dynamics is often used to analyze the wind field changes of UAVs in flight. Through the numerical simulation method, the three-dimensional CFD model and two-phase flow model were established to study the influence of the downwash wind field of the plant protection UAV on the movement trajectory and distribution of droplets (Junfeng et al., 2017; Fengbo et al., 2018; Hao et al., 2019; Juan et al., 2019; Guo et al., 2020). However, in these previous numerical simulation studies, the complex structure of nozzle model is not accurate enough due to the physical model of nozzle in the numerical simulation. The physical model in numerical simulation can not completely represent the real nozzle structure. Nonetheless, all these studies provide specific references and guidance for the study of wind field simulation of plant protection UAVs.

For wind field distribution models under complex conditions, due to the inaccuracy of the physical model and the weak computational force, many simulations will simplify the physical model and reduce the mesh density, resulting in some deviations. For example, in the simulation of the rotor wind field, the structure will be simplified, and the mesh density will be reduced so that the motion state in the rotor wind field can be obtained conveniently and quickly. The computational fluid dynamics method based on the Lattice Boltzmann method (LBM) has advantages in dealing with the complex model boundary of UAV rotors (Fakhari and Lee, 2015; Sheng et al., 2018). It can accurately deal with problems at both micro and macro scales (Tang et al., 2020a; Zhang et al., 2020; Tang et al., 2021; Wang et al., 2021). In addition, with the development of image processing technology, particle image velocimetry (PIV) has been applied to the analysis of spray under the rotor wind field (Jin et al., 2014; Tang et al., 2020b). However, there are few papers on the combination of numerical simulation and PIV image analysis technology to study the downwash flow state of the rotor wind field of plant protection UAVs under dynamic load.

Therefore, this study is devoted to studying the changes in the rotor wind field distribution of plant protection UAVs under dynamic loads (at different rotor speeds) and focuses on revealing the influence mechanism of rotor wind field on droplet distribution characteristics of plant protection UAVs at different rotor speeds. Because the Lattice Boltzmann method has obvious advantages in dealing with complex boundary conditions and non-stationary moving objects, XFlow software is used to simulate the distribution of the downwash wind field of the quadrotor plant protection UAV at different rotor speeds. At the same time, because PIV has the characteristics of non-contact, high measurement accuracy and fast processing speed, PIV is used in this study to measure the spray changes of the wind field under the rotor at different speeds. Through the combination of the two, the conditions of the downwash wind field and the velocity and vorticity of droplets under different rotor speeds are compared. It is hoped that this study can help researchers better understand the distribution characteristics of rotor wind field at different rotor speeds and further reveal the distribution characteristics and rules of droplet deposition under the influence of rotor wind field in the dynamic load state of the quadrotor plant protection UAV.

## Materials and methods

### Numerical simulation

#### Physical model

**Figure 1:**
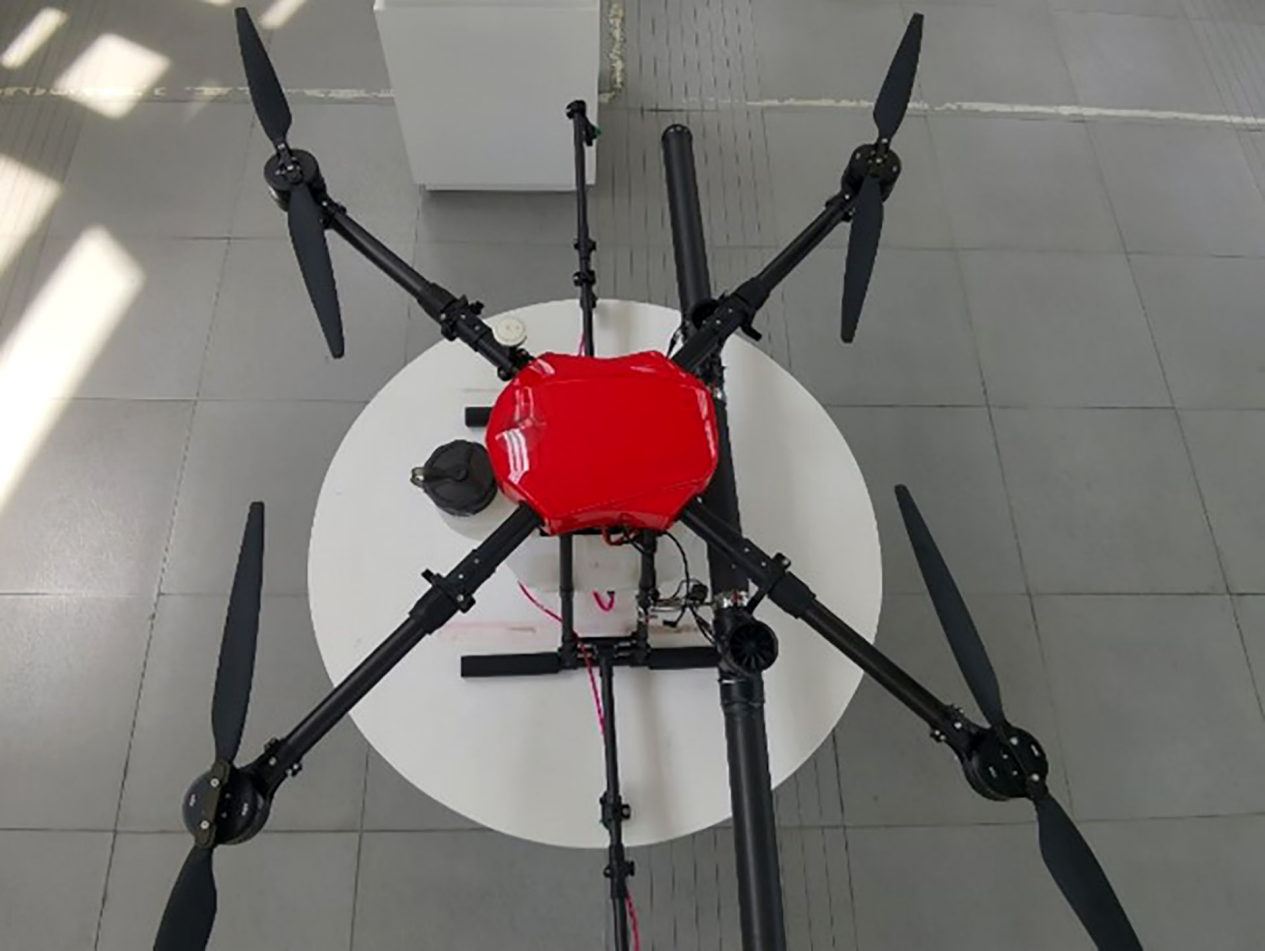
Quadrotor plant protection UAV.

This paper takes the 410S quadrotor plant protection UAV (Xiamen Land and Air Technology Co., LTD.) as the research object. As one of the most representative models in the market at present, the UAV has the functions of manual or semi-automatic route flight, continuous spraying at break point, low voltage protection and so on. The expansion size is 1075×1075×490mm. The folding size is 635×666×490mm, the UAV empty weight is about 5kg (excluding the spraying system), the maximum takeoff weight is 25kg, the rotor size is 30 inches, and the spraying is 1.3-2 acres per sortie. The UAV is shown in **Figure 1**, and its main parameters are shown in **Table 1**.

**Table 1:** Parameters of the quadrotor plant protection UAV.

Parameters	Technical index
**Rotor diameter/m**	1.4
**Typical application speed/m·s^-1^**	3-8
**Rotor speed/rpm**	0-3000
**Load capacity/kg**	10
**Flight duration/min**	8-10
**Operate temperature/°C**	-25-50

The detailed parameters of the Xiamen Land and Air Technology Co., LTD. 410S quadrotor plant protection UAV are listed in **Table 1**.

**Figure 2:**
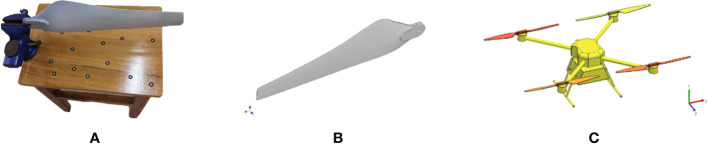
Three-dimensional model of quadrotor plant protection UAV.

The UAV rotor is the essential component to generate the rotor wind field, so it is essential to establish accurate 3D modelling for it. Therefore, in order to accurately simulate the wind field characteristics of the quadrotor UAV at different rotor speeds, it is necessary to conduct a three-dimensional reverse reconstruction of the rotor to establish the physical model of the rotor. In this paper, a handheld 3D scanner N700 (CREAFORM INC.) is used to scan the rotors in three dimensions to obtain the point cloud data of each scanning surface of the rotors, as shown in **Figure 2A**. Then Geomagic Studio software (Geomagic INC.) is used to post-process each scanned surface point and reconstruct the three-dimensional surface model of the rotor, as shown in **Figure 2B**. The body, landing gear and other components of the quadrotor UAV are based on surveying and mapping technology dimensions. Autodesk Inventor Profession (Autodesk INC.) is used to establish a three-dimensional model. The complete three-dimensional model of the constructed electric quadrotor plant protection UAV is shown in **Figure 2C**.

#### Simulation calculation

Commonly used commercial Computational Fluid Dynamic (CFD) software, such as Fluent (ANSYS Inc.) and CFX (ANSYS Inc.), the dynamic mesh technique is usually used in dynamic simulation to analyse the hydrodynamic characteristics of rotors in a high-speed rotating motion. However, for complex quadrotor model boundary cases, the mesh reconstruction in the numerical simulation process usually consumes a large amount of computational time, and it is easy to produce negative volume in the calculation process, leading to calculation errors. XFlow (Next Limit Dynamics S.L.) is a fluid Dynamics simulation and analysis software based on the Lattice Boltzmann method (LBM), which does not need to mesh the model. It has advantages in solving complex boundary conditions and three-dimensional flow field problems of non-stationary moving objects. It can conveniently deal with fluid problems at micro and macro scales. Because this particle-based calculation method does not require traditional grid division and has high efficiency in the discretisation stage and accurate calculation results, this paper adopts the LBM-based XFlow software to simulate the downwash wind field of the quadrotor plant protection UAV.

#### Simulation method

**Figure 3:**
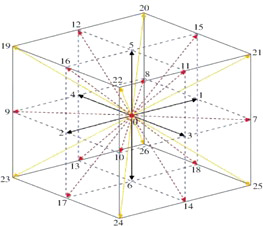
D3Q27 lattice model.

Xflow uses the LBM method, where the computation domain is a uniform cube cell. The LBM is a mesoscopic method, and the macroscopic Navier-Stokers equations can be derived from the lattice Boltzmann equation according to the Chapman-Enskog expansion. In many of the LBM models, XFlow adopts a three-dimensional lattice structure as shown in **Figure 3**, which includes 27 velocity vector directions (D3Q27), 1 discrete velocity vector lattice body to zero point in the centre, 6 discrete velocity vectors from the body of the heart to the centre of the lattice decent, 12 discrete velocity vector from body centre to lattice body midpoint, 8 discrete velocity vectors from the centre of the body to the top Angle of the lattice. Therefore, compared with the traditional LBM, there are higher-order spatial discretisation modes.

In this method, the lattice-Boltzmann equation is selected as the solution equation, and the lattice-Boltzmann transport equation is discretized on the lattice as


(1) $$\begin{array}{c} f_{i}\left(x+e_{i},t+dt\right)=f_{i}\left(x,t\right)+W_{i}\left(x,t\right) \end{array}$$

(2) $$\begin{array}{c} W_{i}=\frac{1}{\tau }\left(f_{i}-f_{i}^{e}\right) \end{array}$$

Where *f_i_* ——Particle velocity vector distribution function; *e_i_* ——The velocity of the particle in the i direction; *d_t_*——Time step; *f_i_(x,t)* ——The velocity distribution function of the particle in i the direction at x point at t time; *w_i_*—— Collision operator; *f_i_^e^*—— One particle equilibrium distribution function; *τ* ——Dimensionless relaxation parameter.

After the approximate simplification of the collision operator, the equation is reduced to the Navier-Stokers equation, which is the governing equation to describe the fluid flow, and the fluid state with a low Mach number can be displayed.

#### Boundary conditions

**Figure 4:**
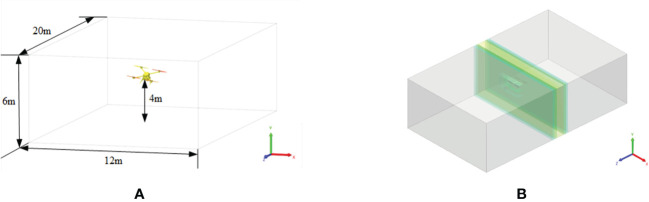
Fluid calculation domain and Discrete division of the computational domain.

The fluid calculation domain set by simulation is a cuboid with a space size of 12m×6m×20m. In the calculation domain, the height of the quadrotor plant protection UAV from the ground is 4m, as shown in **Figure 4A**.

By improving the spatial and temporal resolution of the calculation domain, the flow field data parameters of the rotor can be obtained more accurately to obtain the omnidirectional irregular flow turbulence scale. Since the simulation of rotor plant protection UAV focuses on the rotor surface, fuselage surface and the wake of the downwash wind field of the UAV, the global spatial refinement resolution size of the virtual wind tunnel is set to 0.2m to save computing resources and shorten computing time. The surface refinement method of the fuselage, main rotor and tail is set as an adaptive method, and the resolution of the fuselage and rotor is set as 0.05m. In order to further demonstrate the characteristics of the wake of the downwash wind field, the refinement space domain is set as 12m×6m×1m at the centre of the rotor, and the refinement resolution of the wake of the downwash wind field is set as 0.025m. After the parameters are set, the automatic discretisation effect diagram of numerical simulation is shown in **Figure 4B**. The motion characteristics of the four rotors are set to be rigid and rotate around the Y-axis of their respective coordinate systems.

In order to obtain the movement law of the quadrotor UAVs in the downwash field, numerical simulation analysis is carried out for the quadrotor UAVs in hovering state at different speeds of 1000rpm, 1500rpm, 2000rpm and 2500rpm. Because the rotor of the quadrotor plant protection UAV will produce velocity flow and vortex in the process of rotation, in order to analyse this phenomenon, the simulated phase diagram of two rotors of the quadrotor UAV in a hovering state is selected for analysis.

### PIV experimental

#### UAV system platform

**Figure 5:**
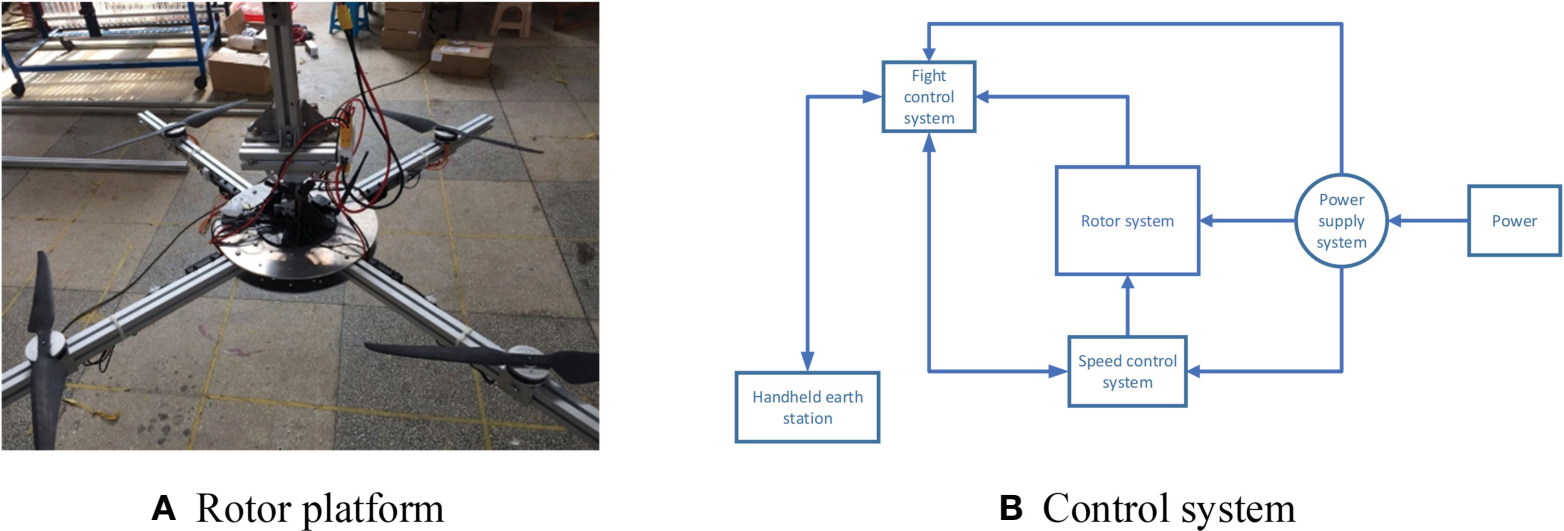
Plant protection UAV test platform.

The UAV rotor used in the test is fixed on the plant protection UAV rotor platform, designed and manufactured by the Nanjing Institute of Agricultural Mechanization. The test platform mainly includes the rotor system, spray system, control system and lifting device. The main body of the test platform is composed of aluminium alloy profiles, which are suspended under the gantry frame. There are three adjustable attitudes ranging from -30°∼30°, which can support using multiple UAVs, such as quadrotor, six-rotor and eight-rotor. The rotor system is specially customized for the test platform. The motor speed of the moving platform is controlled by the ground station software in real-time to achieve the corresponding wind field test effect. At the same time, the parameter information of the platform can be monitored in real time, and the test data can be saved. The quadrotor structure is used in the test, and the height of the rotor is about 2m above the ground. The UAV system platform is shown in **Figure 5**.

Details of power system of plant protection UAV are shown in **Table 2** below:

**Table 2:** Power system parameters of plant protection UAV.

Parameters	Technical index
**Rotor diameter/m**	1.4
**Rotor speed/rpm**	0-3000
**Operation duration time/min**	10-15
**Operate height/m**	1-3

The spray system of the platform is installed next to the gantry frame and consists of a water pump and a control system. The spray system supports the hydraulic nozzle and centrifugal spray nozzle. The pressure range is 0∼1.2*MPa*, the flow range is 0~2*L/min*, and the spray time is automatically controlled. In this experiment, a fan-shaped 110-02 nozzle designed and manufactured by Lechler is used to produce droplets with an average volume diameter of 120*μm*. Because the relative density and size of droplets meet the particle requirements required by the PIV test, the droplets produced by the sprinkler head can be directly used as tracer particles for the PIV test. Meanwhile, the nozzle is installed 35cm below the rotor.

PIV system

**Figure 6:**
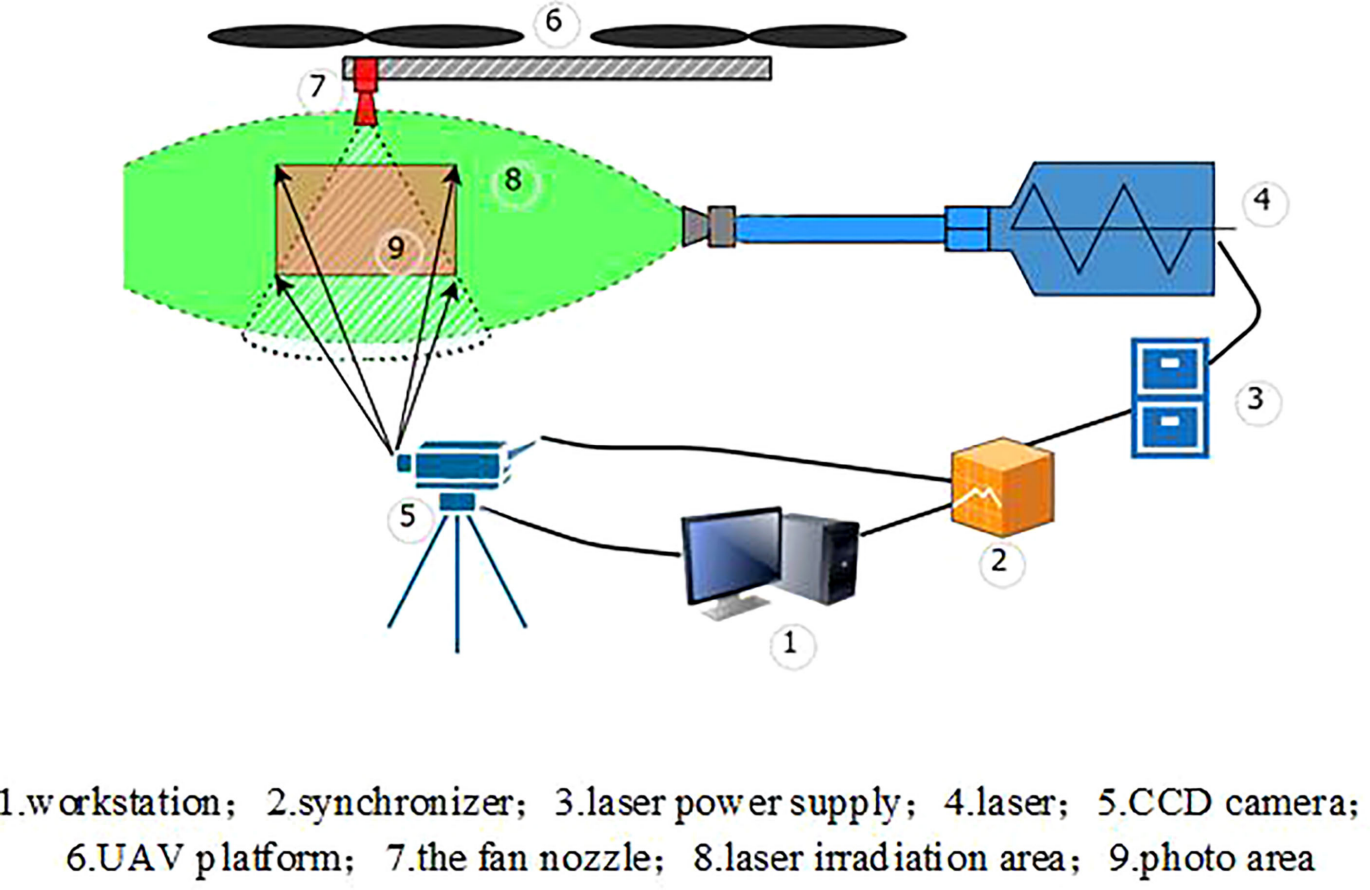
Schematic diagram of the experiment.

Because the rotor vortex caused by the downwash wind field of the quadrotor plant protection UAV is aperiodic, the PIV device is selected as the measurement tool to measure the fluid domain. PIV technology is a transient, multi-point, non-contact fluid dynamics measurement method, which can record the velocity distribution information of particles at a space point at the same time, provide abundant spatial structure and flow characteristics of the flow field, and has very high measurement accuracy. The PIV system consists of high-speed cameras (2048*2048, 32 FPS; TSI Incorporated, USA), a pulsed laser (380 mJ/pulse, wavelength = 532 nm; TSI Incorporated, USA), etc. The laser generated by the pulsed laser is combined through an optical system composed of a cylindrical mirror and a spherical mirror to generate a slice light source with a waist thickness of about 1 mm. The frame rate is resized according to the actual situation. In this experiment, the schematic setting of the whole PIV system experiment is shown in **Figure 6**.

**Figure 7:**
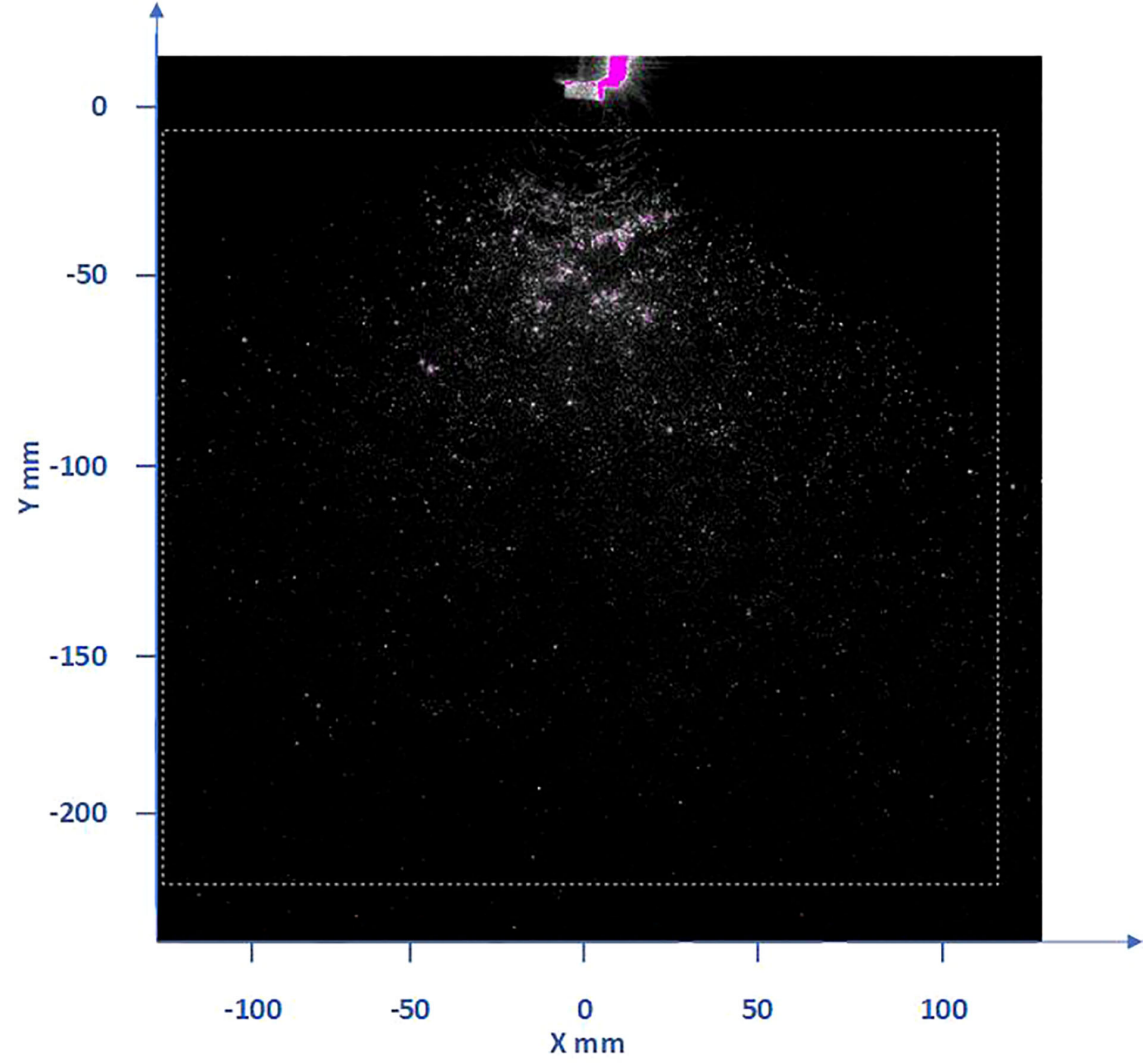
Image of droplets distribution in the downwash flow field of the nozzle under the rotor. (The red particles are the droplets irradiated by the laser, and the dashed box is the set Mask flow field analysis area).

A 50mm lens is used to generate a large enough field of view to capture the motion state of the flow field particles, which is the evolution of particles scattering from the nozzle into the air. The movement of droplet particles in the wind field under different rotor speeds is captured by setting Mask, as shown in **Figure 7**. In this experiment, the pulse width of the YAG laser pulse is 3-5ns, the time interval of two laser beams is 50μs, the time series between two pictures is 0.025s, and the maximum distance of particle movement is less than 1mm. Therefore, the query window is set to 36*36 pixels (a 4.6×4.6mm square) to ensure that the particle moves less than a quarter of the query window length, and the overlap rate is set to 50×50mm.

The results of PIV are obtained by obtaining the average value of flow field data from multiple consecutive double-frame images when the rotor speed is 0, 1000rpm, 1500rpm, 2000rpm and 2500rpm, respectively. Based on its high sampling frequency, the turbulent kinematic energy is calculated from the two position components of the velocity fluctuation as follows:


$$k=\frac{1}{2}\left(u{'}^{2}+v{'}^{2}\right)$$

Where *u* ^′^ is the horizontal component, *v* ^′^ is the vertical component.

Insight 4G software is used to obtain two consecutive instantaneous AB frames spray images, analyze the flow field in the images, and generate the original data. Tecplot software is used to obtain the image data to generate the velocity vector map. The resolution of these images is 2048*2048 pixels. The nozzle is located at (0,0) in the coordinate system, and the rotor is located directly above the nozzle. Because the laser beam irradiates the droplet field from the right and the droplets have a refraction effect, the light on the left side is weaker than that on the right side, so the effect on the right side of the whole droplet field is obviously stronger than that on the left side, but the actual effect should be consistent. According to the rotor speed used in the numerical simulation analysis, the corresponding rotor speed of 1000rpm, 1500rpm, 2000rpm and 2500rpm is also selected for the PIV test to observe the influence of the wind field below the rotor on the droplet velocity flow and vortex in the test. The velocity and vorticity motion of droplets under the action of the wind field is analyzed.

## Results and analysis

### Numerical simulation of rotor flow field

#### Distribution of rotor velocity under numerical simulation

**Figure 8:**
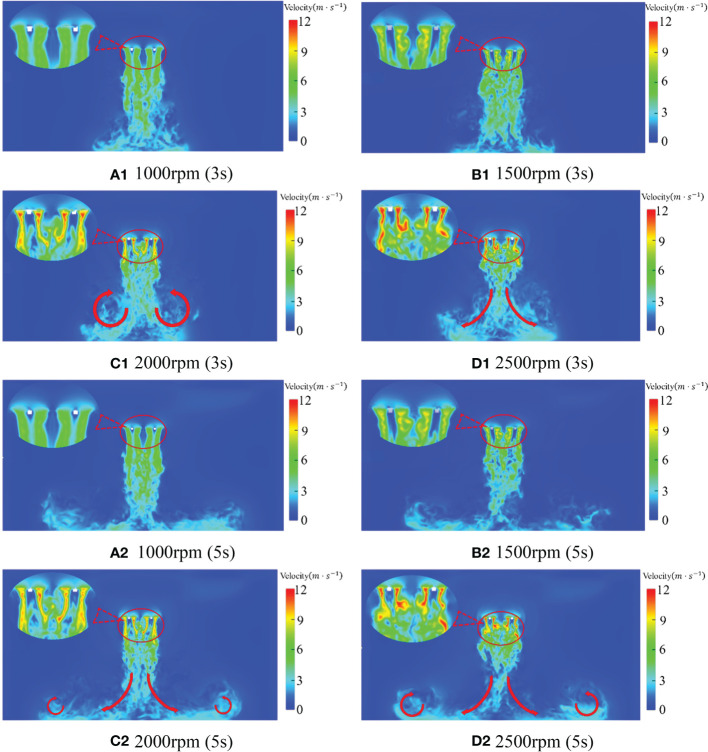
Rotor velocity distribution at different speed and time.

The rotor speeds studied in the test are respectively 1000rpm, 1500rpm, 2000rpm and 2500rpm. Therefore, 3s and 5s at different times are selected to study the distribution of flow fields at different rotor speeds to compare the differences of flow fields. **Figure 8** is the velocity state phase diagram of the downwash wind field at 3s and 5s when the rotor speed of the quadrotor plant protection UAV is 1000rpm, 1500rpm, 2000rpm and 2500rpm, respectively. As can be seen from **Figures 8A1-D1**, with the increase of rotor speed, the speed value under the rotor of the quadrotor UAV gradually increases, with the maximum value increasing from 7.6m/s at 1000rpm to 15.7m/s at 2500rpm. Due to the presence of turbulence, the velocity core area under each rotor (velocity greater than 10m/s) also gradually breaks apart, forming four distinct velocity core areas. At the same time, a very low value of local velocity occurs just below the centre of each rotor in the process of rotor rotation. Furthermore, with the increase of rotor speed, the situation of extremely low local velocity becomes more apparent. By observing **Figure 8A1** and **Figure 8A2**, it is found that when the rotation speed is 1000rpm, the situation of the very low local speed is not apparent, indicating that the quadrotor plant protection UAV is still in the relatively initial state when it is actually 1000rpm and has not reached the stable flight state.

As shown in **Figures 8A2-D2**, with the increase of rotor speed, the maximum speed under the rotor of the quadrotor UAV varies from 7.9m/s at 1000rpm to 14.6m/s at 2500rpm. This shows that the quadrotor plant protection UAV does not reach a stable flight state at 1000rpm while the maximum speed under the rotor decreases at 2500rpm. The reason for the decrease is that the velocity flow field under the rotor gradually diffuses around and becomes more widely distributed after a period of development. In addition, part of the wind bouncing on the ground also rises, which offsets the downwash wind, so the downwash force is weakened, and the maximum speed is reduced(as shown by the red arrow in the figure). By observing **Figure 8**, it can be seen that the contraction distortion of the velocity core area caused by the rotor is not apparent when the rotational speed is 1000rpm and 1500rpm in the figure. However, the contraction distortion of the velocity core area under the rotor is noticeable when the rotor speed is 2000rpm and 2500rpm. It indicates that the quadrotor plant protection UAV basically reaches a stable flight state when the rotor speed is above 2000rpm.

**Figure 9:**
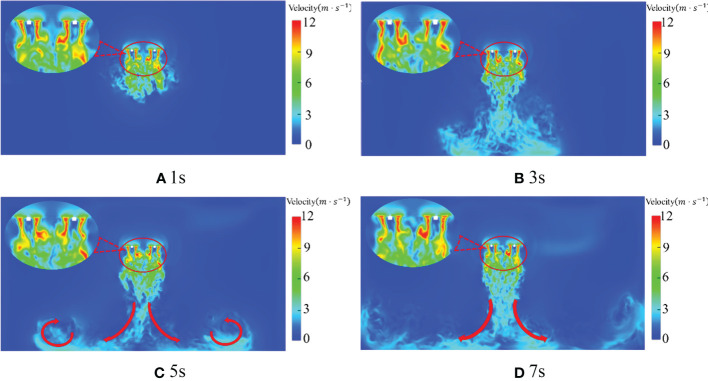
Rotor velocity distribution at a different time and 2500rpm.

Because the flow field has just reached the ground and generated turbulence at 3s and the flow field is formed at 5s when the rotor speed is 2500rpm, the rotor speed is 2500rpm for analysis. **Figure 9** is the speed state phase diagram of the quadrotor plant protection UAV at 1s, 3s, 5s and 7s when the rotor speed is 2500rpm. It can be seen from **Figures 9A-D** that a jellyfish-like wind field with the speed decreasing from the fuselage has been generated around the quadrotor plant protection UAV at 1s. The maximum wind field at each time is 14.8m/s, 15.7m/s, 14.6m/s and 14.9m/s respectively. Thus, it can be seen that the wind field initially acts together in the high-speed area formed above the rotor, integrates with each other, diffuses to the top of the whole fuselage, and rapidly decreases from the fuselage to the surrounding areas. The velocity core area has been formed just below the rotor at 1s. With the gradual downward development of the wind field, the tail is broken more violently in the development process, strong turbulence appears below, and the influence range gradually increases downward. The flow field has reached the ground, and the maximum speed of the wind field also reaches the highest at this moment, which is 15.7m/s at 3s. Then the wind field, due to contact with the ground, collision with the ground, rebound and spread around, a violent turbulent phenomenon occurred(as shown by the red arrow in the figure). The maximum speed gradually reduced, kept below 15 m/s. It can be seen that with the increase of time, the velocity flow field under the quadrotor UAV gradually diffuses downward, and the range of the flow field gradually increases. After bouncing with the ground, the airflow is absorbed by the low pressure generated by the high-speed airflow, and the spiral airflow is gradually formed in the vertical space. Taking the centre line of the fuselage as the axis of symmetry, the flow field is about in the x-direction (-1m, 1m). At the same time, from the perspective of flow field development, the velocity flow field is basically developed and formed around 5s.

According to **Figures 8**, **9**, when the rotor of the quadrotor UAV rotates, the velocity core area generated by the two rotors with opposite rotation directions is basically symmetric, and the overall posture of aggregation, contraction and downward pressure is presented after the development and formation. The local extremely low velocity under the rotor becomes more obvious with the increase of rotor speed. As the distance from the rotor increases, the velocity core area generated by the downwash wind field under the rotor gradually decreases, and the distance of about 1m below the rotor basically disappears.

#### Distribution of rotor vorticity under numerical simulation

**Figure 10:**
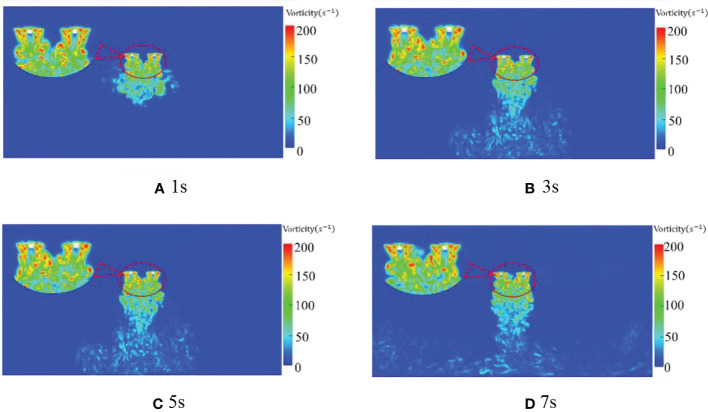
Distribution of rotor vorticity at different times and 2500 rpm.

**Figure 10** is the cross-sectional phase diagram of the vortex state of the quadrotor plant protection UAV at the hover time of 1s, 3s, 5s and 7s when the rotor speed is 2500rpm. First of all, it can be seen from **Figure 10** that the maximum vorticity at each moment reaches 337s^-1^, 376*s* ^-1^, 304*s* ^-1^ and 343*s* ^-1^ respectively, showing that the maximum vorticity rises first and then decreases. The maximum vorticity around the rotor has reached 337*s* ^-1^ at 1s, and the vortex on the rotor surface is concentrated and distributed around the rotor. Second, the vortex moves down in a vertical direction under each rotor. Due to the contraction distortion characteristic of the wingtip vortex, the contraction effect is induced by the following wake vortex. After leaving the rotor surface, the vortex gradually shrinks. Under the coupling effect of the vortex, the rotor vortex is damaged and eventually forms turbulence. As shown in **Figure 10**, with the fuselage centre line as the axis of symmetry, the main vortex field is about in the x-direction (-1m, 1m).

With the passage of time, the maximum vorticity reaches 376*s* ^-1^ at 3s. Then the larger value of vorticity begins to decline and mainly concentrates within 0.5m below the rotor. At the same time, the rest parts gradually move further below the rotor and contain larger vorticity. Larger vorticity may even appear within 2m below the rotor and then be destroyed by coupling, resulting in turbulence. However, the vorticity beyond 2m below the rotor is small and basically exists in the form of turbulence. This indicates that the quadrotor plant protection UAV produce a strong enrolling effect within 1m below the rotor and the enrolling effect is weaker, followed by 1m-2m. At the same time, in the vertical direction, the spiral vortex decreases with the increase of the distance from the rotor.

**Figure 11:**
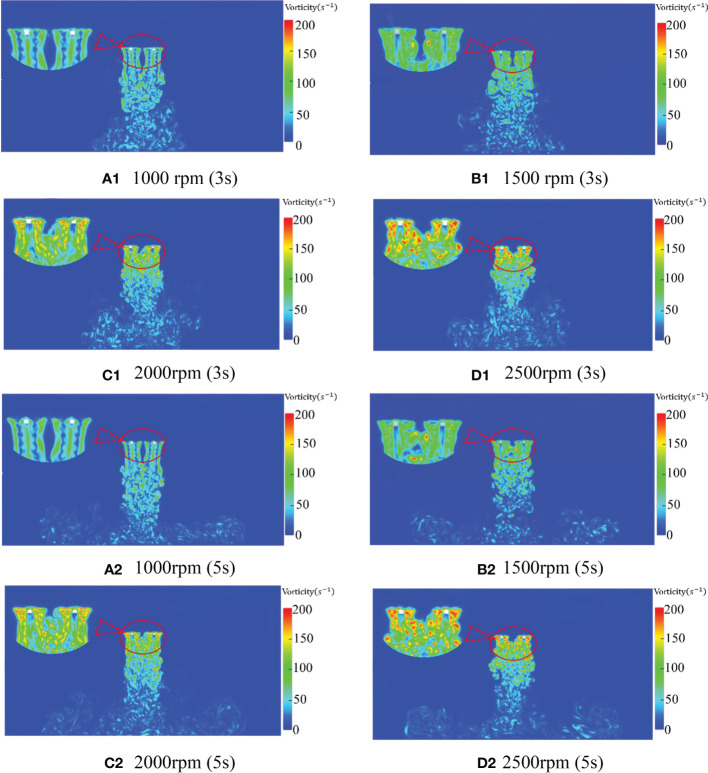
Distribution of rotor vorticity at different speeds and different times.

Since the velocity field at 3s and 5s time is mainly observed and analyzed in the wind field analysis, and a similar flow field also appears in the vortex field, this paper also analyze the vortex at various speeds at 3s and 5s time. **Figure 11** is the vortex state phase diagram of the downwash wind field at 3s and 5s when the rotor speed of the quadrotor plant protection UAV is 1000rpm, 1500rpm, 2000rpm and 2500rpm, respectively. As shown in **Figures 11A1-D1**, the vorticity also increases, and the vortex changes more violently with the increase of rotor speed. In **Figure 11A**, the vortex within 1m below the rotor shows apparent symmetry, showing a relatively stable state as a whole. In the range of 1m-2m below the rotor, relatively chaotic turbulence is generated due to the coupling effect of the rotor vortex. In the range of 2m to 4m below the rotor, the turbulent effect is more apparent, and the vortex has a strong irregular movement. At the same time, the symmetrical vortex generated by the rotor decreases obviously with the increase of rotor speed. When the time is 3s, the symmetric vortices with the rotation speed of 1000rpm, 1500rpm, 2000rpm, and 2500rpm mainly appear within 1m, 0.8m, 0.6m, and 0.5m below the rotor, respectively. The maximum vorticity under the rotor also changes from 216*s* ^-1^ at 1000rpm to 376*s* ^-1^ at 2500rpm with the increase of rotor speed.

Although part of the vortex symmetry is still maintained in **Figure 11A2** compared with **Figure 11A2**, the maximum vorticity under the rotor is 213*s* ^-1^ in **Figure 11B2**, and the vortex is greatly disorganized. In **Figure 11B2**, the vortex under the rotor even appears to have temporary disconnection. As seen from **Figure 11B1**, the maximum vorticity becomes 170*s* ^-1^ with the increase of time at 1000rpm. This indicates that the vortex of the quadrotor plant protection UAV developed with time at this speed appeared disorder after reaching the ground at 3s. By observing the vortex state of 1500rpm, 2000rpm and 2500rpm at 5s, the maximum vorticity of 1500rpm and 2000rpm at 5s is 246*s* ^-1^ and 283*s* ^-1^, respectively, which also shows a certain degree of disorder, but the degree of influence gradually decreases. The vortex state at 2500rpm appears more stable and develops more stably than 3s. By comparison, it can be seen that the greater the rotor speed, the longer the vortex needs to reach relative stability.

According to **Figures 10**, **11**, when the rotor of the quadrotor UAV rotates, the vortices generated by the two rotors with opposite rotation directions are basically symmetric, and the overall posture of aggregation, contraction and downward is presented after the development and formation. Moreover, the greater the rotational speed, the longer it takes for the vortex to reach a relatively stable state. At the same time, with the increase of the distance from the rotor, the vorticity gradually decreases, and the enrolling effect of the larger vortex group is mainly concentrated within 1m below the rotor.

### PIV experiment of the rotor flow field

#### Distribution of rotor velocity under the PIV experiment

**Figure 12:**
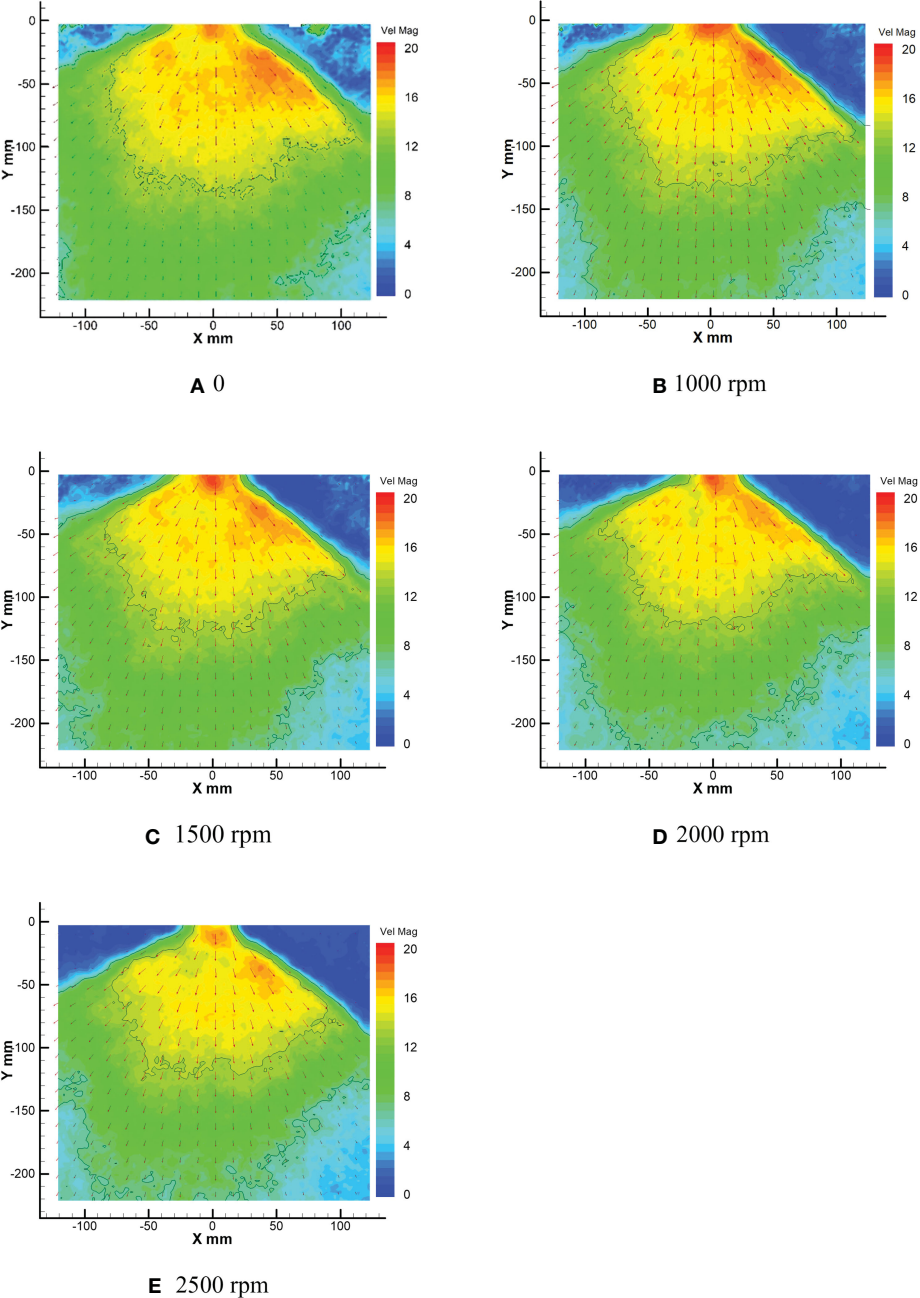
Average droplet velocity field at different rotor speeds.

As shown in **Figure 12**, they are the droplet velocity fields at rotor speeds of 0, 1000rpm, 1500rpm, 2000rpm and 2500rpm, respectively. **Figure 12A** shows that the expanding state of droplets is mainly distributed in the sector. The effective range of droplets greater than 16 m/s (defined as high-speed) in the sector area is mainly distributed in the sector area of X (-70, 100) Y (0, -150), while the regional velocity in other areas is mainly in the range of 6-16 m/s (defined as medium-speed). The droplet velocity in the lower right corner is mainly below 6 m/s (defined as low-speed), which has been shown by curves in the figure of each area. There are medium-speed droplets in the air in the upper left and upper right corners and much turbulence. As the speed increases to 1000rpm (**Figure 12B**), compared with the wind field without downward pressure in **Figure 12A**, under the influence of the downwash wind field generated by the rotor, the speed and range of action of the droplets in the sector area have changed significantly. The turbulence pattern in the upper left and upper right corners shows a particularly significant change. The number of droplets decreases, and the velocity decreases significantly to almost zero. Under the nozzle, not only the velocity value but also the high-speed range of the droplets increases significantly. The high-speed area near the nozzle is shaped as a half ellipse with a long axis perpendicular to the Y-axis. The fan distribution of droplets generated by the nozzle is more obvious. The effective range of the high-speed area of droplets is mainly distributed in the fan area of X (-70, 90) Y (0, -140), the effective range of the medium-speed area decreases, and the low-speed area in the lower left and right corners increases. On the edge of both sides of the sector area, there are apparent channels of medium-speed droplets layer between the high-speed area and the air.

It can be seen from **Figures 12C-E** that with the increase of rotor speed, the downward pressure wind field is gradually enhanced, and the droplet high-speed area under the rotor contracts, obviously. Not only does the high-speed zone contract, but the droplet velocity decreases gradually. The effective range distribution of the high-speed zone gradually contracts from X (-65, 90) Y (0, -130) to the sector of X (-55, 85) Y (0, -110). In addition, the core area of the high-speed zone below the nozzle is the most obvious situation: the droplet area and velocity decrease. Even the core area is mostly disconnected from the surrounding area of the high-speed area (**Figure 12E**). At the same time, the low-speed area in the lower left corner and lower right corner of the area in the figure gradually increases with the increase of rotor speed, especially the expansion in the lower right corner is the most obvious.

By comparing the conditions in the high-speed area of each droplet velocity field in **Figure 12**, it can be found that the droplet velocity in the central area of the sector in the high-speed area tends to decrease successively. This trend not only indicates that the number of droplets is more concentrated in the unit volume, which causes the laser coming from the right side to be refracted more and weakens the intensity of the laser on the left side but also indicates that under the action of the wind field under the rotor, the speed of the droplets emitted from the fan nozzle weaken with the increase of the rotor speed. The expansion of the low-speed zone between the lower left corner and the lower right corner also proves that the droplet velocity from the fan nozzle weakens with the increase of rotor speed.

**Figure 13:**
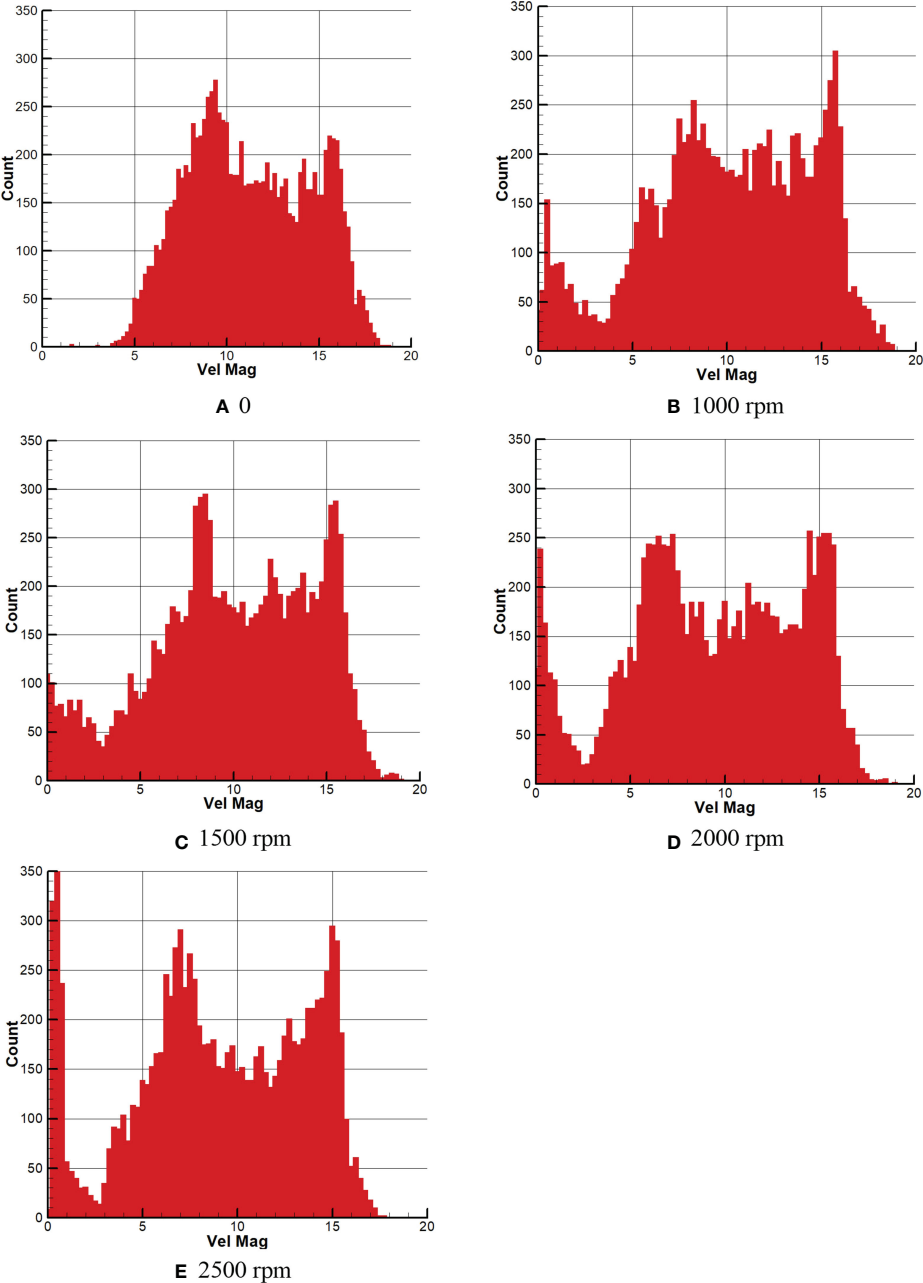
Quantitative distribution of average droplet velocity at different rotor speeds.

Similarly, **Figure 13** shows the number distribution of droplets in different rotor speed intervals. **Figure 13A** shows that the maximum droplet velocity is about 18 m/s, and there are mainly two peaks in the quantity distribution of droplet velocity. The peak with the most significant number occurs in the interval of 8.5-9.5 m/s, followed by the interval of 15.5-16.5 m/s. The speed of droplets is primarily concentrated in the medium speed interval of 6-16 m/s, followed by high speed and low speed. **Figure 13B** shows the distribution of droplet velocity when the rotor speed is 1000rpm, and the maximum droplet velocity is about 19 m/s. With the appearance of the downwash wind field under the rotor, the maximum droplet velocity does not increase significantly, but the peak value of the number distribution of droplet velocity changes. However, the peak value of the medium-speed area becomes more. At the same time, a large number of low-speed droplets also appears in the low-speed area where there are only a few droplets, and the peak value is generated. The peak value of the medium-speed area is mainly distributed in the interval of 7-9 m/s, 11-12 m/s, 13.5-14.5 m/s, and 15-16 m/s, while the peak value of the low-speed area is mainly in the interval of 0.5-1.5 m/s.

**Figures 13C-E** show the quantity distribution of droplet velocity at 1500rpm, 2000rpm and 2500rpm of rotor speed, respectively. It can be seen from the figures of 13c and 13d that when the rotor speed is 1500rpm and 2000rpm, the maximum speed of the droplet field is basically 19m/s. However, in **Figure 13E**, when the rotor speed is 2500rpm, the maximum speed of the droplet field is only 18m/s, indicating that the droplet field is affected by the increase of the rotor speed. The maximum velocity of the droplet field is also weakened. At the same time, compared with **Figure 13B**, the total droplet velocity in the high-speed area decreases, and two apparent peaks appear in the medium-speed area. In addition, by comparing the medium-speed intervals of **Figures 13C-E**, it can be found that the peak value of the medium-speed zone extends from the intervals of 8-9 m/s and 15-16 m/s in **Figure 13C** to 6.5-8 m/s and 13.5-16 m/s, respectively, indicating that the number of droplets in the medium-speed zone gradually increases and the effect of wind field downward pressure is obvious. At the same time, it can also be seen from the figures of the three, the number of droplets in the low-speed area also increases significantly. The number of droplets in the low-speed area with the speed toward zero concentration is the most obvious, especially in **Figure 13E**.

As seen in **Figure 13**, the speed in the overall droplet field is weakened due to the generation of the rotor wind field. Furthermore, with the increase of rotor speed, it can be seen that the droplet velocity in the low-speed area gradually concentrated at 0, and the turbulence in the whole study area basically disappear. Due to laser refraction caused by the concentration of the number of droplets on the right side, the droplets in the left area are not sufficiently obtained, and the velocity data are not apparent. Because of this, the peak value of the total number of droplets in the medium-speed area gradually tends to both sides of the medium-speed area, which makes two prominent peaks appear in the medium-speed area, which should be relatively gentle.

In conclusion, it can be seen from **Figure 12** that the high-speed area generated at the nozzle location is obviously different due to the downwash wind field caused by the increase of rotor speed. With the increase of rotor speed, the downwash wind field is gradually strengthened, and the high-speed area at the nozzle is gradually contracted and decreased. At the same time, the total spray angle of the nozzle is gradually reduced, and the number of droplets in the sector area is gradually concentrated. The high-speed area gradually decreases, and the low-speed area gradually expands in the whole spray sector area. As can be seen from **Figure 13**, the maximum velocity of droplets in the sector area is 19 m/s, which is basically unchanged. When the total number of droplets in the high-speed area is 1000rpm, the number of droplets in the high-speed area is the largest, and the number of droplets in the high-speed area gradually decreases with the increase of the rotor speed. The number of droplets in the medium-speed zone and low-speed zone increases obviously. The turbulent conditions in the upper left and upper right corners disappear.

#### Distribution of rotor vorticity under the PIV experiment

**Figure 14:**
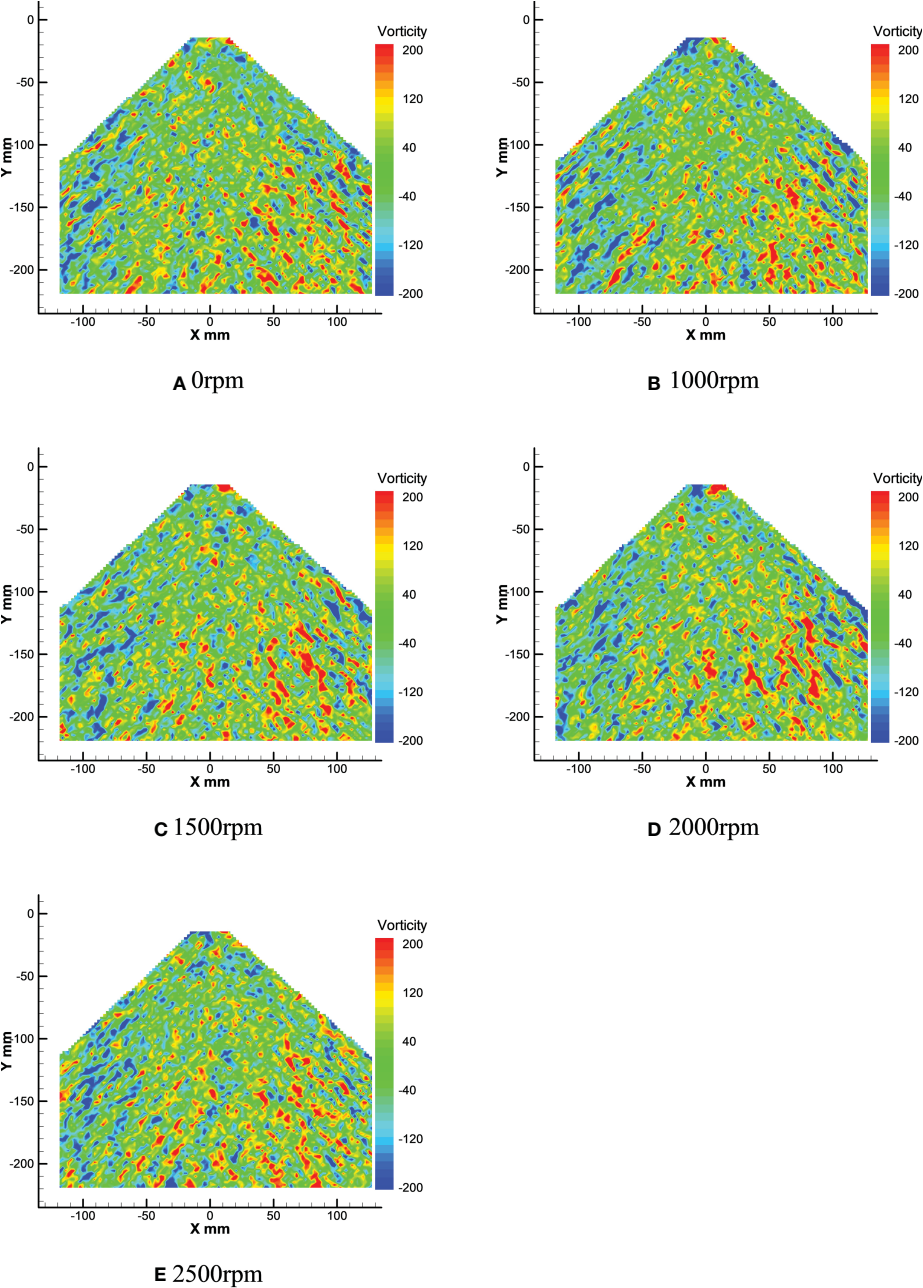
Average droplets vorticity field at different rotor speeds.

**Figure 15:**
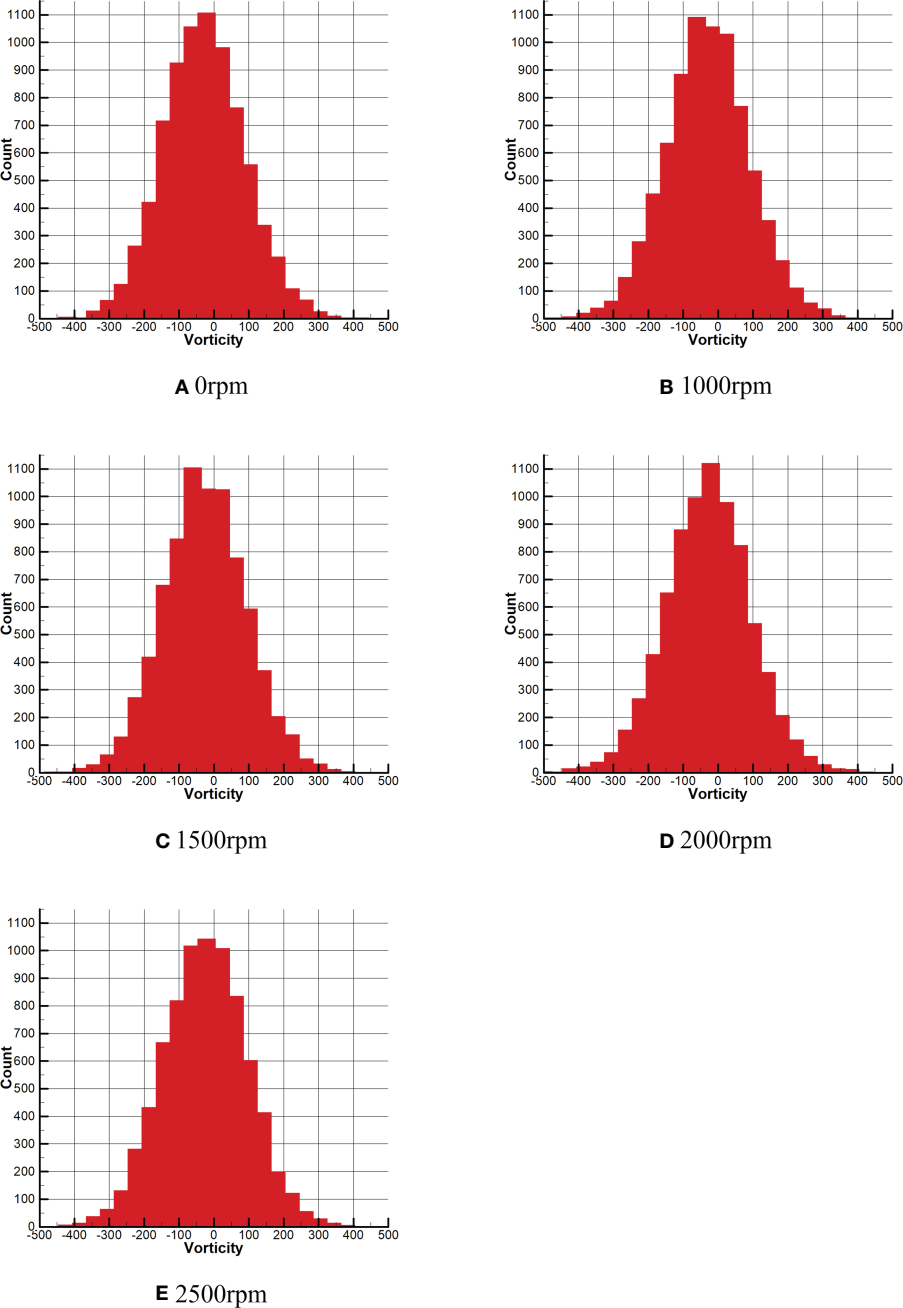
Quantity distribution of the average droplet vorticity at different rotor speeds.

**Figure 14** shows the vortex field of the nozzle droplet at different rotor speeds. **Figure 15** shows droplet vortex size and quantity distribution at different rotor speeds.

Through the vortex analysis of **Figure 14**, it can be seen that positive vortex and negative vortex cross in the droplets field, and it can be clearly seen that the left side of the droplets field is dominated by negative vortex, and the right side is dominated by positive vortex. Through numerical analysis of the vortex field, it can be seen that in **Figure 14A**, the extreme values of the vortex in the droplets field are -449.14*s* ^-1^ and 819.02*s* ^-1^ respectively. However, under the effect of the rotor wind field, the extreme value here changes. In **Figure 14B**, the extreme value of the vortex in the droplets field changes to -502.91*s* ^-1^ and 463.75*s* ^-1^. Compared with **Figure 14B**, the positive vortex in the droplets field has significantly changed and decreased by 355.27*s* ^-1^, while the negative vortex in the droplets field has slightly changed and only increased by 53.77*s* ^-1^. In **Figures 14C**, **14D**, the extreme value of the negative vortex in the droplets field decreases, both of which are around -485*s* ^-1^. The extreme value of the positive vortex in **Figure 14C** reaches 589.64*s* ^-1^, while the extreme value of the positive vortex in **Figure 14D** reaches 478.32*s* ^-1^, decreasing by 111.44*s* ^-1^. In **Figure 14E**, the extreme values of the droplet field are -597.93*s* ^-1^ and 463.27*s* ^-1^ respectively. Compared with **Figure 14D**, the positive vortex in the droplets field decreased by 15.05*s* ^-1^, while the negative vortex in the droplet field changed greatly and increased by 113.51*s* ^-1^.

The vortex between (-500, 500) are selected as the X-axis, and the number of vortex under different vorticity is selected as the Y-axis for analysis in the **Figure 15**. As can be seen from **Figure 15A**, the total number of vortex in the range of (-100, 0) exceeds 1000. After the rotor wind field is generated, as shown in **Figure 15B**, the range of the total number of vortex exceeding 1000 changes to (-100, 50), and the range is expanded. In **Figure 15C**, the range where the total number of vortex exceeds 1000 remains basically unchanged. However, it can be seen from **Figure 15D** that the range of the total vortex exceeding 1000 becomes smaller, and only the range (-50, 0) exceeding 1000. However, from the whole of **Figure 15D**, the droplets are more concentrated on both sides of the 0 vortex. In **Figure 15E**, the range of vortex exceeding 1000 is mainly concentrated in the range (-100, 50), but the total number of vortex in this range is somewhat reduced compared with that in **Figure 15B**.

According to the combination of **Figure 14** and **Figure 15**, the left side of the droplets field is dominated by negative vortex, while the right side is dominated by positive vortex. With the increase of rotor speed, the extreme value and number of vortex in the droplets field change in different degrees. The negative vortex in the droplets field increases first, then decreases and then increases, while the positive vortex decreases first, then increases and then decreases. Compared with the vortex under the effect of no wind field, the maximum value of negative vortex under the effect of rotor wind field is reduced by 148.79*s* ^-1^, while the maximum value of positive vortex is reduced by 355.27*s* ^-1^. Meanwhile, it can be seen from **Figure 15** that under the effect of no wind field, the majority of vortex near 0 in the vortex field are negative vortex. However, with the increase of rotor speed, the number of positive vortex near 0 and negative vortex is gradually equal.

## Discussion

**Figure 16:**
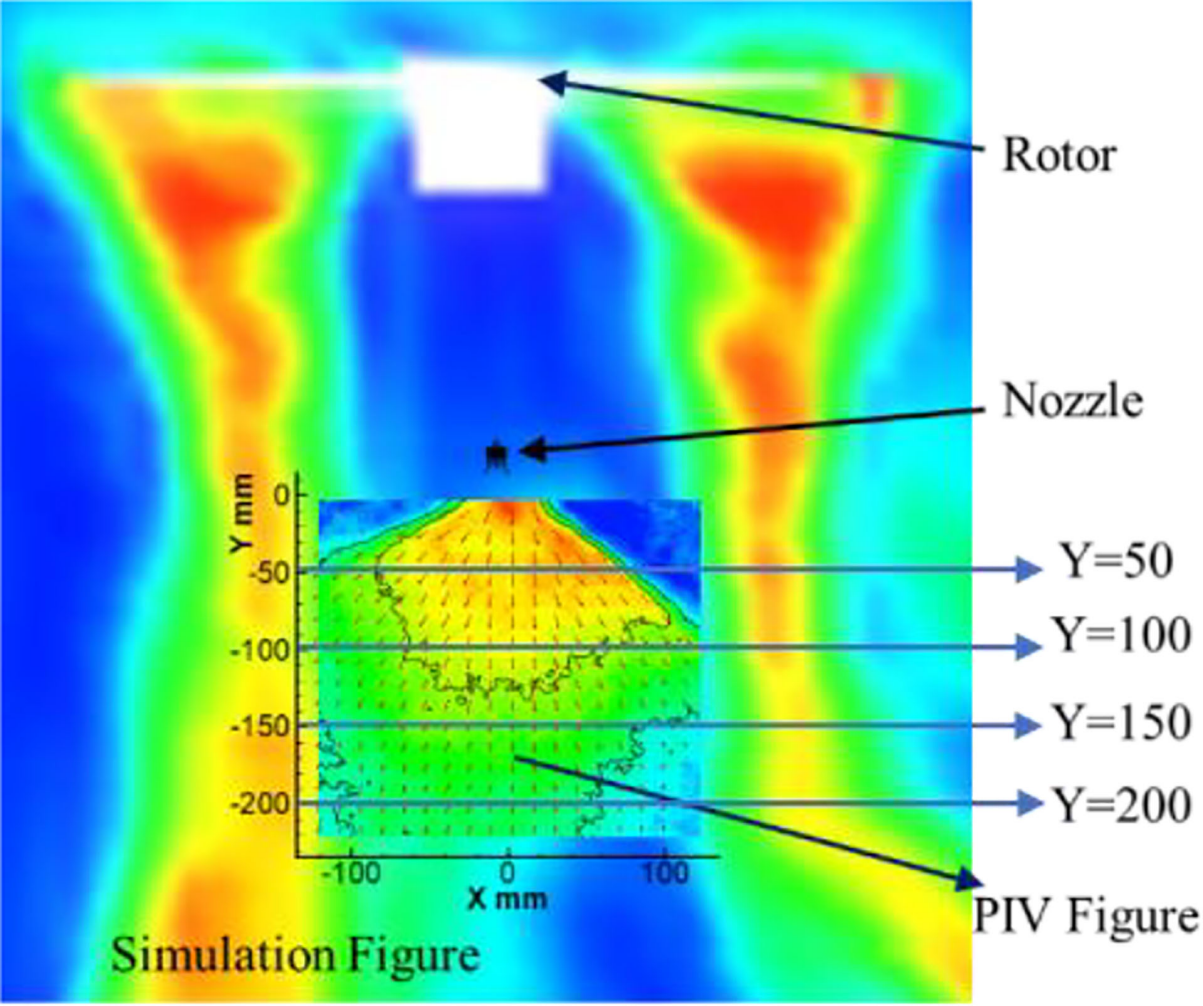
The schematic diagram.

**Figure 17:**
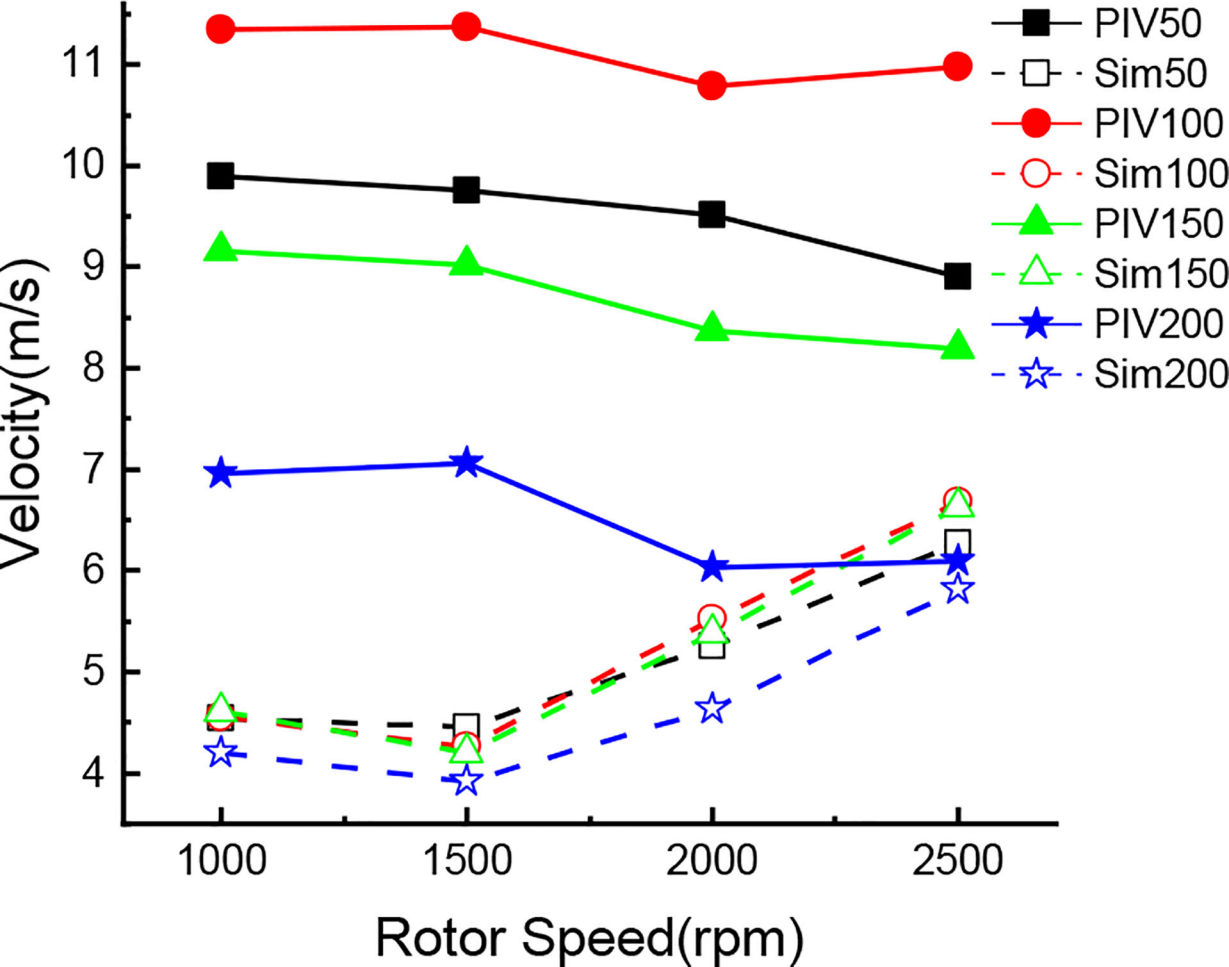
The change trend of the mean value of PIV and numerical simulation.

In this paper, the droplet velocity distribution at four different horizontal levels (Y=50mm, 100mm, 150mm, 200mm) of the coordinate system in PIV image is selected for comparative analysis and research with wind field velocity at four different horizontal levels corresponding to the coordinate system in numerical simulation. The schematic diagram of horizontal level selection is shown in **Figure 16**. In this study, the data under each level are averaged to obtain the average value. In the numerical simulation test, the data at the time of 3s is selected for analysis. In the data of PIV experiment, the vertical velocity data is selected as the analysis sample. Thus, the mean value and variance results of PIV test and numerical simulation are shown in **Table 3**, and the change trend of the mean value of PIV and numerical simulation is shown in **Figure 17**.

**Table 3:** The mean value and variance results of PIV test and numerical simulation.

	Rotor Speed/rpm	Y=50mm		Y=100mm		Y=150mm		Y=200mm	
		Mean	SD	Mean	SD	Mean	SD	Mean	SD
PIV	1000	9.9	5.32	11.35	2.65	9.17	2.56	6.96	1.74
	1500	9.75	5.31	11.38	2.61	9.03	2.61	7.06	1.68
	2000	9.53	5.22	11.8	2.93	8.37	2.51	6.03	1.43
	2500	8.91	5.50	10.99	2.84	8.19	2.61	6.1	1.53
Sim	1000	4.54	1.91	4.56	1.79	4.61	1.82	4.20	1.86
	1500	4.45	2.24	4.26	1.99	4.20	1.86	3.92	1.72
	2000	5.25	2.70	5.52	2.95	5.38	3.30	4.63	3.35
	2500	6.27	2.35	6.68	2.75	6.63	1.86	5.81	2.30

As can be seen from the **Figure 17**, with the increase of rotor speed, the average velocity of the wind field in the numerical simulation test shows an overall trend of increase, while the average velocity of droplets in the PIV test shows an overall trend of decrease, indicating that the wind field significantly reduces the average velocity of droplets. As can be seen from the **Figure 17**, in the numerical simulation test, the wind speed of four levels at each rotor speed has little difference.

However, in the PIV test, there is a large difference in droplet velocity under different levels. The average velocity of droplets at the level of Y=200 mm is the minimum, while the average velocity of droplets at the level of Y=100 mm is the maximum, even exceeding the average velocity of droplets at the level of Y=50 mm. This is because at the level of Y=50mm, the velocity of some droplets is close to 0 and the velocity variance is about 5.3, while at other levels, the velocity variance of droplets is about 2.6, thus dragging down the overall average velocity.

Meanwhile, it can also be seen from **Figure 17** and **Table 3** that the wind speed generated by the wind field increases gradually on the whole with the gradual increase of rotor speed. However, when the rotor speed is 1000rpm, the speed in the wind field is higher than that when the rotor speed is 1500rpm. According to the analysis results of the speed value, the variance of the speed value is smaller than that of other speeds when the rotor speed is 1000rpm. This indicates that when the rotator speed is 1000rpm, the vortex in this range is small and the velocity value is relatively uniform. Combined with the PIV test results, it can be seen that the variances of PIV droplet speeds at these two speeds are also very close, indicating that the PIV test data can effectively reflect the accuracy of the numerical simulation. When the rotor speed in the numerical simulation exceeds 2000rpm, the droplet velocity in the PIV test decreases significantly, and the rotor downwash wind field has a significant influence on the movement velocity of droplets at all levels. Although the speed at the level of Y=200mm in numerical simulation is the smallest compared with other levels, combined with the change of droplets in PIV test, it has a certain influence on the droplets at the level of Y=200mm. Combined with the above analysis, it can be seen that this is because the numerical simulation of the wind field at Y=200mm began to appear a certain degree of vortex, which reduced the movement speed of droplets.

Through the above analysis, it can be seen that in the numerical simulation, the speed is relatively stable at 1000rpm and 1500rpm. Under these two rotor speeds, the droplet velocities at all levels in PIV are also basically stable, which proves that the droplet velocity under PIV test can effectively verify the reliability of the numerical simulation. At the same time, when the rotor speed exceeds 2000rpm, the speed in the numerical simulation increases significantly. Correspondingly, the droplet velocity in PIV test all decreased to varying degrees, among which the droplet velocity change was most obvious at the level of Y=200mm. Therefore, combined with the above analysis, PIV test can effectively verify the validity of the numerical simulation results.

At present, the mechanical analysis of droplet velocity and vorticity variation has not been effectively and comprehensively verified. In this paper, PIV test is used to effectively analyze the motion state of droplets field under the action of wind field. By analyzing the distribution of velocity flow and vortex in droplet field, it is concluded that under the action of wind field of rotor, the velocity of droplet is reduced, while the vortex shrinks and the vorticity increases. PIV test can reflect the reliability of numerical simulation results from the side. The downwash wind field of plant protection UAV is very complicated, and it has a very important influence on the deposition and drift of droplet. Therefore, based on the actual operation of plant protection UAVs, this paper expounds the influence mechanism of rotor wind field of plant protection UAVs on droplets distribution characteristics under different rotor speeds based on the distribution characteristics of rotor wind field under dynamic load (at different speeds), providing references for researchers in this field.

## Conclusion

The XFlow software was used to simulate the downwash wind field of the quadrotor plant protection UAV at different rotor speeds, and the particle image velocimetry (PIV) was used to measure the motion state of droplets at different rotor speeds. The main findings of this experiment are summarized as follows:

The experimental results of numerical simulation show that the maximum velocity and vorticity of the downwash field under the rotor increase with the increase of rotor speed. However, with the increase of time, turbulence is generated, and the maximum values of the downwash wind velocity and vorticity decrease. The velocity flow field under the rotor becomes more widely distributed. In addition, the velocity flow and vortex under the rotor are symmetrically distributed in the centre line of the fuselage, mainly distributed in the range of (-1m, 1m) in the X direction. The larger value of velocity flow is mainly concentrated in the area within 1m below the rotor, and the vortex is mainly concentrated in the area within 0.5m.

The results of the PIV test show that with the increase of rotor speed, the total spray angle and the high-speed area of the spray area gradually shrink and decrease under the action of the downwash wind field, while the low-speed area in the spray area gradually expands. In addition, the maximum velocity of droplet particles under the rotor wind field is 19m/s. The number of droplet particles decreases gradually in the high-speed area, while the number of droplet particles increases gradually in the medium-speed area and low-speed area. When there is no downwash wind field, there is a lot of turbulence in the fan droplet area, and the maximum vorticity is the 819.02. But under the effect of the downwash wind field produced by the rotor, the vortex is contracted. Under the effect of no wind field, most of the eddies near 0 are negative vortex in the vortex field, but with the increase of rotor speed, the number of positive vortex near 0 and negative vortex is gradually equal.

Through comparative analysis of the PIV test and numerical simulation results, it can be seen that the maximum speed of the numerical simulation wind field in the area within 0.5m below the rotor reaches 15.7m/s. In the PIV test, the speed of the droplet in the droplet field in this range is about 18 m/s under the action of the nozzle pressure, and the speed of the turbulent droplet in the upper left and upper right corner outside the sector area is about 8m/s. Due to the increase of rotor speed and the enhancement of the downwash wind field, the turbulence disappears in the upper left and upper right corners of the sector area, and the number of low-speed droplets increases in the lower left and right corners of the sector area in the PIV test, which indicates that the PIV test results effectively verify the reliability of the numerical simulation results.

## Data availability statement

The original contributions presented in the study are included in the article/supplementary material. Further inquiries can be directed to the corresponding authors.

## Author contributions

KC, SC, and YL: conceptualization. KC: formal analysis and writing-original draft. SC, MW and YL: resources and supervision. SC, XX and YL writing-review and editing and funding acquisition. YL: project administration. All authors contributed to the article and approved the submitted version.
